# Artificial neural network retrained to detect myocardial ischemia using a Japanese multicenter database

**DOI:** 10.1007/s12149-018-1247-y

**Published:** 2018-03-07

**Authors:** Kenichi Nakajima, Koichi Okuda, Satoru Watanabe, Shinro Matsuo, Seigo Kinuya, Karin Toth, Lars Edenbrandt

**Affiliations:** 1 Department of Nuclear Medicine, Kanazawa University Hospital, 13-1 Takara-machi, Kanazawa, 920-8641 Japan; 2 Department of Physics, Kanazawa Medical University, Uchinada, Kahoku, Japan; 3 EXINI Diagnostics, Lund, Sweden; 4 Department of Clinical Physiology and Nuclear Medicine, University of Gothenburg, Gothenburg, Sweden

**Keywords:** Nuclear cardiology, Artificial intelligence, Myocardial perfusion imaging, Coronary artery disease

## Abstract

**Purpose:**

An artificial neural network (ANN) has been applied to detect myocardial perfusion defects and ischemia. The present study compares the diagnostic accuracy of a more recent ANN version (1.1) with the initial version 1.0.

**Methods:**

We examined 106 patients (age, 77 ± 10 years) with coronary angiographic findings, comprising multi-vessel disease (≥ 50% stenosis) (52%) or old myocardial infarction (27%), or who had undergone coronary revascularization (30%). The ANN versions 1.0 and 1.1 were trained in Sweden (*n* = 1051) and Japan (*n* = 1001), respectively, using ^99m^Tc-methoxyisobutylisonitrile myocardial perfusion images. The ANN probabilities (from 0.0 to 1.0) of stress defects and ischemia were calculated in candidate regions of abnormalities. The diagnostic accuracy was compared using receiver-operating characteristics (ROC) analysis and the calculated area under the ROC curve (AUC) using expert interpretation as the gold standard.

**Results:**

Although the AUC for stress defects was 0.95 and 0.93 (*p* = 0.27) for versions 1.1 and 1.0, respectively, that for detecting ischemia was significantly improved in version 1.1 (*p* = 0.0055): AUC 0.96 for version 1.1 (sensitivity 87%, specificity 96%) vs. 0.89 for version 1.0 (sensitivity 78%, specificity 97%). The improvement in the AUC shown by version 1.1 was also significant for patients with neither coronary revascularization nor old myocardial infarction (*p* = 0.0093): AUC = 0.98 for version 1.1 (sensitivity 88%, specificity 100%) and 0.88 for version 1.0 (sensitivity 76%, specificity 100%). Intermediate ANN probability between 0.1 and 0.7 was more often calculated by version 1.1 compared with version 1.0, which contributed to the improved diagnostic accuracy. The diagnostic accuracy of the new version was also improved in patients with either single-vessel disease or no stenosis (*n* = 47; AUC, 0.81 vs. 0.66 vs. *p* = 0.0060) when coronary stenosis was used as a gold standard.

**Conclusion:**

The diagnostic ability of the ANN version 1.1 was improved by retraining using the Japanese database, particularly for identifying ischemia.

## Introduction

The diagnostic ability of artificial neural network (ANN), which is a type of artificial intelligence, has been examined from the viewpoint of nuclear cardiology applications [1, 2]. A multicenter study was the first in Japan to apply an ANN to myocardial perfusion imaging (MPI) during 2015 [3]. That ANN was trained to detect myocardial stress perfusion defects and induced ischemia on a Swedish database, but its diagnostic ability was comparable to that of expert interpretation for Japanese patients. Thereafter, the diagnostic ability was further improved by training the ANN on a Japanese multicenter database (*n* = 1,001) using ^99m^Tc-methoxyisobutylisonitrile (MIBI) MPI [4]. That validation study indicated that the ANN had good diagnostic ability comparable to nuclear cardiology expert interpretation, as the area under the receiver-operating characteristics (ROC) curve (AUC) was 0.92.

However, whether or not the diagnostic accuracy of version 1.1 actually improved from the initial cardioREPO software version 1.0 (FUJIFILM RI Pharma Co. Ltd., EXINI Diagnostics, Lund, Sweden) has not been validated. In addition, the conditions under which the diagnostic ability of version 1.1 changed have remained unknown. The present study aimed to determine whether the diagnostic ability of version 1.1 trained on a Japanese database was improved over the original version by comparison with the same population that was used before [3].

## Methods

### Participants

The participants were as described for the validation study of the first version (cardioREPO version 1.0) [3]. A total of 106 patients (male, 61%; mean age, 70 ± 10 years) who underwent coronary angiography within 1 month of MPI were selected from Public Central Hospital of Matto Ishikawa, Kanazawa Cardiovascular Hospital, and Kanazawa University Hospital. When the number of vessels with coronary stenosis ≥ 50% was defined as abnormal, 25, 29, 30 and 22 patients had 0, 1-, 2-, and 3-vessel disease (total of patients with multi-vessel disease: 52%). Comorbidities comprised hypertension (58%), diabetes mellitus (33%) and dyslipidemia (36%), and 27 and 30% of the patients had a clinical history of old myocardial infarction and coronary revascularization, respectively. All clinical data were completely anonymized and processed at Kanazawa University. The Ethics Committee at Kanazawa University approved the study.

### Myocardial perfusion imaging and diagnosis

Patients were assessed using a stress–rest sequence with a standard dose (maximum, 1,110 MBq) and a protocol for ^99m^Tc perfusion tracers [5]. Acquired energy was centered at a ^99m^Tc window of 140 keV ± 10%. Stress was imposed either by exercise (89%) or pharmacologically using adenosine (11%). Electrocardiographic gating on the dual-headed SPECT system was 16 frames per cardiac cycle. Attenuation and scatter correction were not applied.

Left ventricular ejection fraction and volumes were also calculated [6, 7].

The final diagnoses of ischemia or infarction were the same as those in the first report [3]. Briefly, a diagnosis was concluded based on the consensus of three experienced nuclear medicine physicians similar to clinical diagnostic procedures to determine ischemia. Original short-axis images and polar maps were presented with information only about age and sex. Left ventricular function, including volumes and ejection fraction, was then added, and all subsequent information about coronary artery stenosis, restenosis, and location of stents or bypass grafts was added. Therefore, the presence of a stress abnormality and of stress-induced ischemia was determined based on the integrated understanding of coronary stenosis and the presence of infarction.

### Artificial neural network training

The first version of the ANN was trained on data from 1,051 Swedish patients (male: 47%; age, 62 ± 10 years) and experienced Swedish physicians classified perfusion as normal or defective [2]. Twelve hospitals in Japan collaborated to train version 1.1 (*n* = 1,001 patients; 75% male; 69 ± 10 years) using ^99m^Tc-MIBI as the tracer [4]. At least two Japanese nuclear cardiology experts determined abnormal stress defects and stress-induced ischemia by consensus. Areas of possible perfusion abnormalities in stress and rest images (stress and rest defects, respectively) were segmented, and the ANN judged candidate regions in terms of the probability of abnormalities (ANN probability) based on 16 features extracted from the shape, extent, location, count, perfusion homogeneity, regional motion, wall thickening and sex.

### Defect scoring

Scoring was based on a 17-segment model [8] and a 5-point scale (0, normal; 1, slight decrease; 2, moderate decrease; 3, severe decrease; 4, complete defect) and calculated automatically by the cardioREPO software (version 1.1). Summed stress (SSS), summed rest (SRS) and summed difference (SDS) scores were included. Defect severity was classified using the database of the Japanese Society of Nuclear Medicine working group that included normal stress–rest findings on SPECT images that were acquired using an Anger camera and not attenuation-corrected [9, 10].

### Statistics

Data are shown as means ± standard deviation (SD). Differences between groups were assessed using a one-way analysis of variance, Student’s *T* tests and *F* tests, and areas under ROC curves were calculated using JMP version 12 (SAS Institute Inc., Cary, NC, USA) statistics software. The appropriate threshold values for sensitivity and specificity were determined at the point at which the maximum sensitivity + specificity − 1 was obtained. A significant difference was indicated when *p* < 0.05.

## Results

**Fig. 1:**
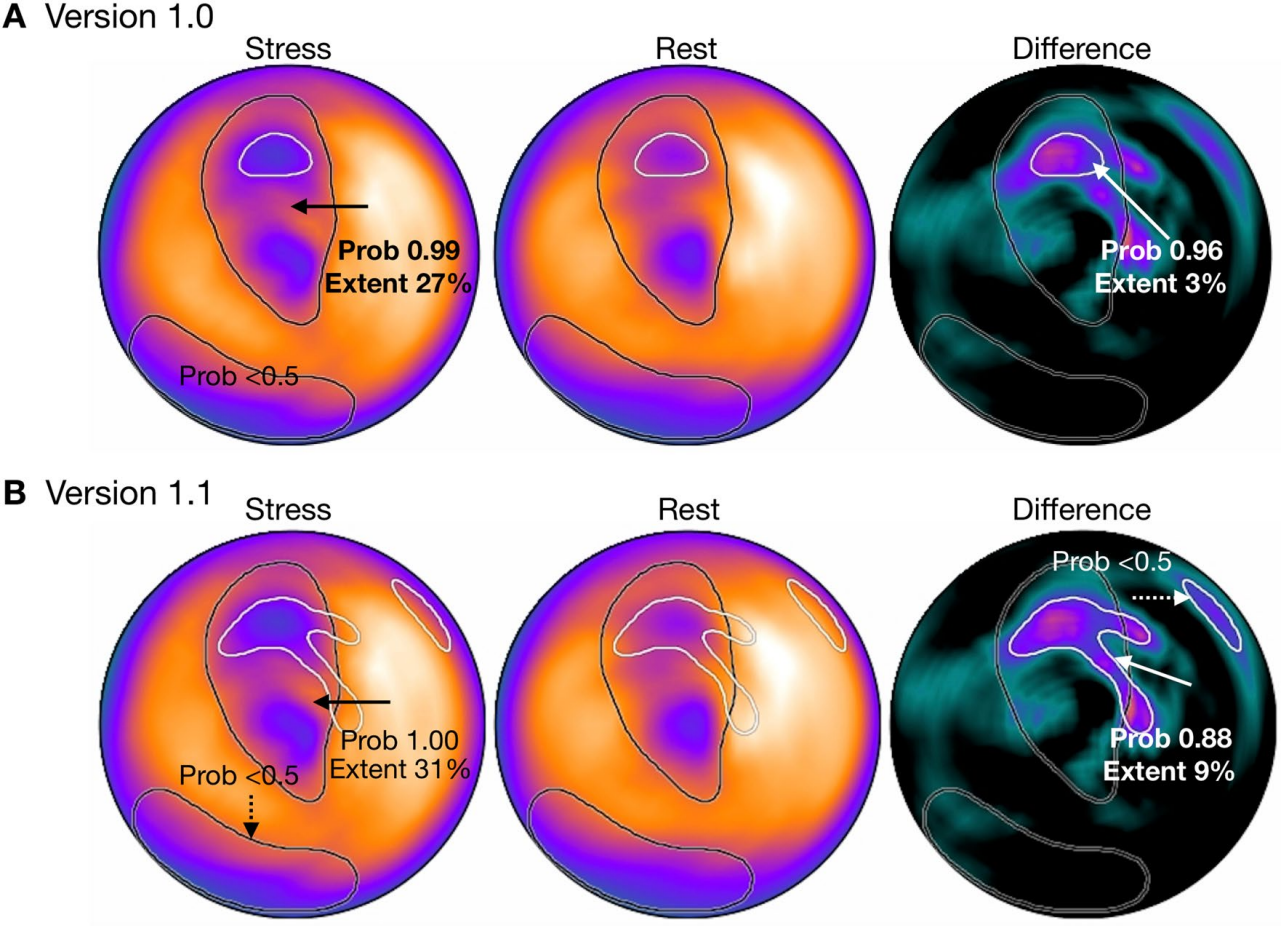
Comparison of segments of abnormality between versions 1.0 and 1.1. ANN probability (Prob) and extent (%) are shown as stress and difference polar maps

Figure 1 shows differences in the segmentation of abnormal regions between versions 1.0 and 1.1 in a patient with anterior myocardial infarction accompanied by exercise-induced ischemia. The area of ischemia was small (probability, 0.96; extent, 3%) in version 1.0. A larger area with a probability of 0.88 and an extent of 9% was identified in the anterior wall, but a small basal region that was selected as candidate was determined as insignificant (probability, < 0.5).

Stress defects and induced ischemia were compared between ANN probability determined by both software versions and expert interpretation (Table 1). The ANN probability values for patients with and without stress defects were 0.87 ± 0.21 and 0.25 ± 0.34, respectively (*F* ratio, 134; *p* < 0.0001), with version 1.0, and 0.85 ± 0.21 and 0.23 ± 0.28, respectively, with version 1.1 (*F* ratio 170; *p* < 0.0001). Values for patients with and without stress-induced ischemia were 0.70 ± 0.40 and 0.01 ± 0.10 (*F* ratio 152; *p* < 0.0001) with version 1.0, and 0.79 ± 0.20 and 0.21 ± 0.22 with version 1.1 (*F* ratio, 195; *p* < 0.0001), respectively.

**Table 1:** ANN probabilities and expert interpretation by versions 1.0 and 1.1

	*N*	Mean	SD	Lower 95% of mean	Upper 95% of mean	*F* ratio	*p*
A. Stress defect							
Version 1.0							
No stress defect	37	0.25	0.34	0.13	0.36	134	< 0.0001
Stress defect	69	0.87	0.21	0.82	0.92		
Version 1.1							
No stress defect	37	0.23	0.28	0.14	0.32	170	< 0.0001
Stress defect	69	0.85	0.21	0.80	0.91		
B. Induced ischemia							
Version 1.0							
No ischemia	55	0.01	0.10	− 0.01	0.04	152	< 0.0001
Ischemia	51	0.70	0.40	0.59	0.82		
Version 1.1							
No ischemia	55	0.21	0.22	0.15	0.27	195	< 0.0001
Ischemia	51	0.79	0.20	0.73	0.85		

*ANN* artificial neural network, *SD* standard deviation

**Fig. 2:**
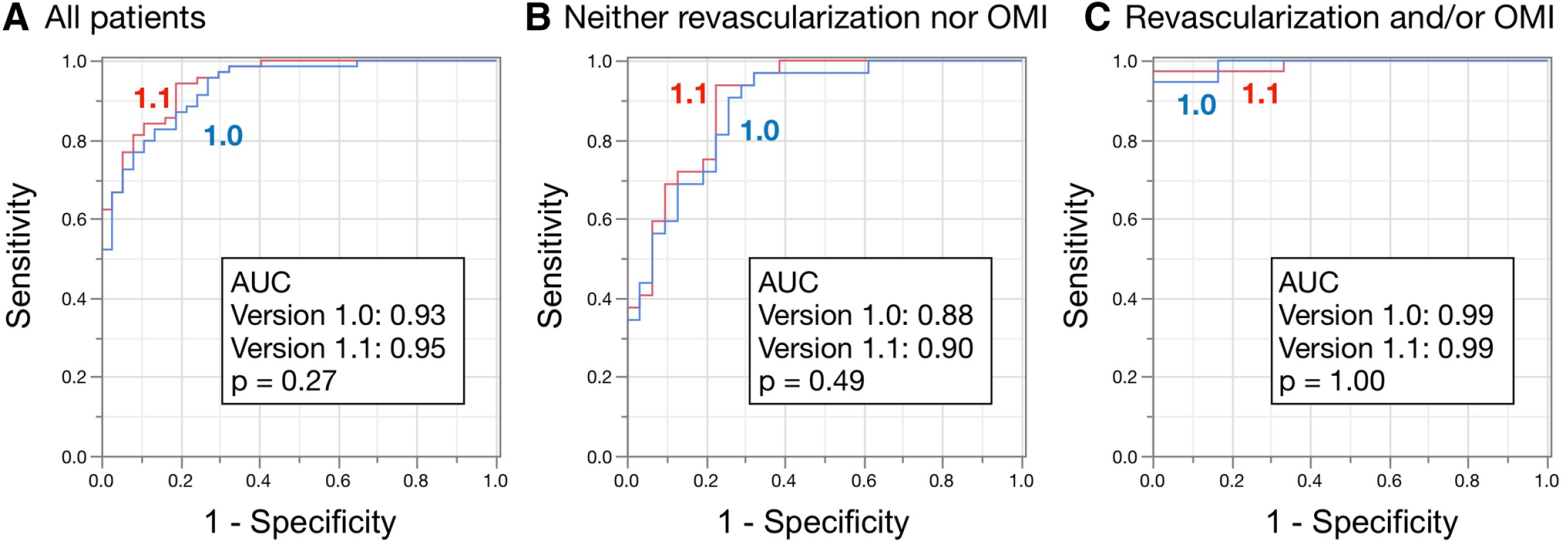
Area under ROC curves to analyze stress defects in all patients (**a**). Without either revascularization or OMI (*n* = 47) (**b**). With revascularization and/or OMI (**c**) (*n* = 59). The statistical measures of sensitivity/specificity/accuracy were 83/86/84% and 94/81/90% for versions 1.0 and 1.1, respectively, for all patients (**a**); 91/74/83% and 94/77/86% for patients without either revascularization or OMI (**b**); and 95/100/95% and 97/100/98% for patients with revascularization and/or OMI (**c**). *OMI* old myocardial infarction, *ROC* receiver-operating characteristics

**Fig. 3:**
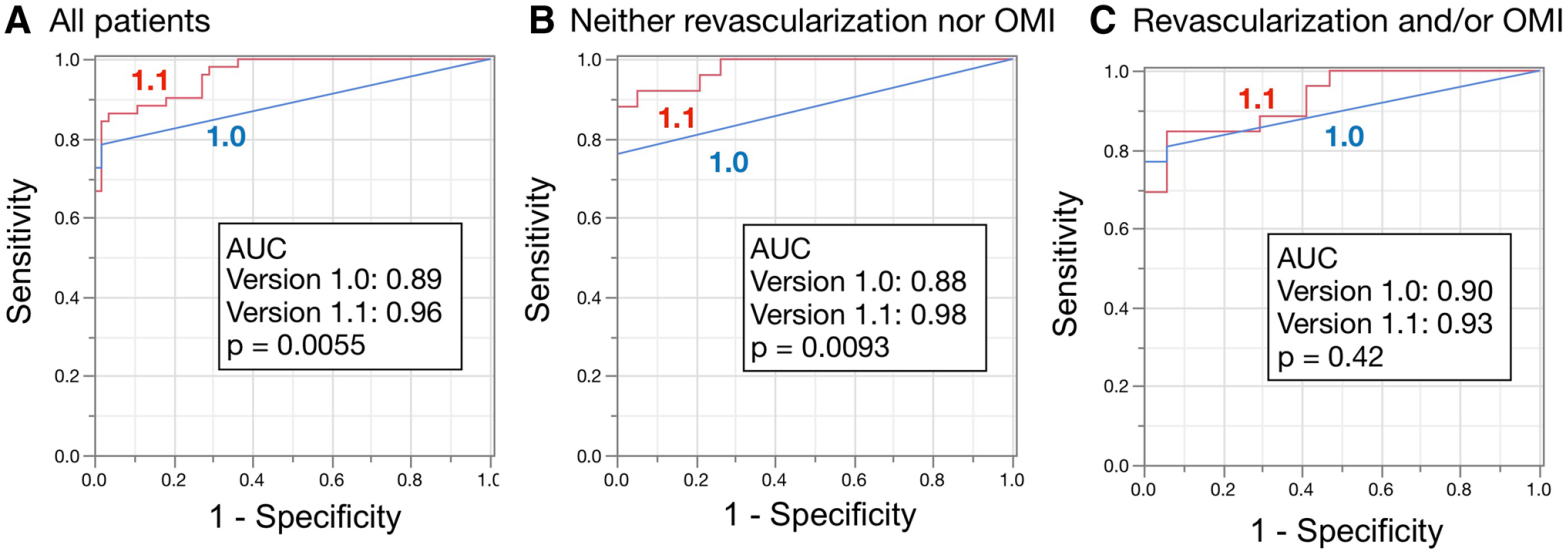
Area under ROC curves to analyze induced ischemia. All patients (**a**), without either revascularization or OMI (*n* = 47) (**b**); with revascularization and/or OMI (*n* = 59) (**c**). The statistical measures of sensitivity/specificity/accuracy were 78/98/89% and 87/96/92% for versions 1.0 and 1.1, respectively, for all patients (**a**); 76/100/90% and 88/100/95% for patients without either revascularization or OMI (**b**); and 77/100/86% and 85/94/88% for patients with revascularization and/or OMI (**c**). *OMI* old myocardial infarction, *ROC* receiver-operating characteristics

Figures 2 and 3 show the results of ROC analyses for detecting stress defects and induced ischemia, respectively, and statistical measures of sensitivity, specificity, and accuracy. Figure 2 shows that the AUC for detecting stress defects calculated by versions 1.0 and 1.1 were 0.93 and 0.95, respectively, which did not significantly differ (*p* = 0.27). The AUC did not significantly differ (*p* = 0.49 and 1.00) even when patients were divided into groups without either revascularization or old myocardial infarction (OMI), and with revascularization and/or OMI. In contrast, Fig. 3 shows that the AUC for ischemia was better for version 1.1 (0.96) than for version 1.0 (0.89, *p* = 0.0055). The AUC was better for version 1.1 (0.98) than for version 1.0 (0.88, *p* = 0.0093) when patients had neither revascularization nor OMI, but did not differ significantly between those with revascularization and/or OMI (*p* = 0.42). Using the version 1.1 sensitivity and specificity for all patients were 94 and 81%, respectively, with stress defect, and 87 and 96%, respectively, with stress–rest difference.

**Fig. 4:**
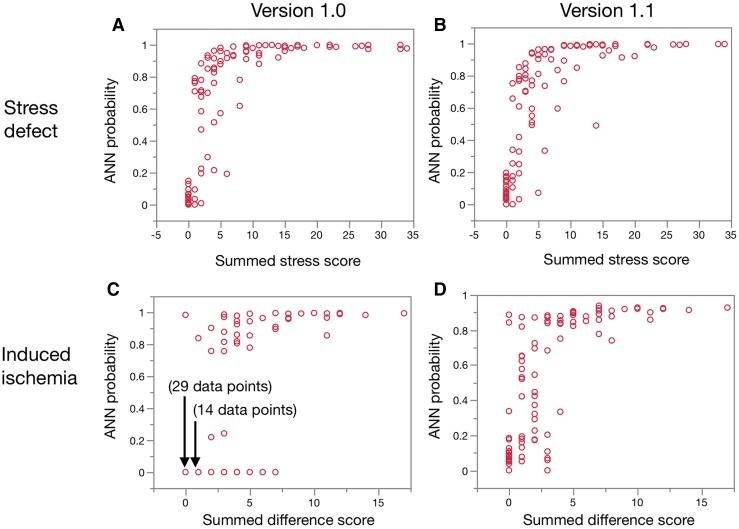
Relationships between ANN probability and summed stress and summed difference scores between two software versions. Summed stress (**a, b**) and summed difference (**c, d**) scores for versions 1.0 and 1.1, respectively

Figure 4 shows the relationship between ANN probability and summed scores. The ANN probability steeply increased in the range of SSS 0 to 5 for both software versions. With respect to induced ischemia, although many points of data overlapped at an SDS of 0–1 and an ANN probability of 0 in version 1.0, ANN probability scattered in a low SDS range of 0–4, indicating a higher prevalence of intermediate ANN probabilities in a range between 0.1 and 0.7.

**Fig. 5:**
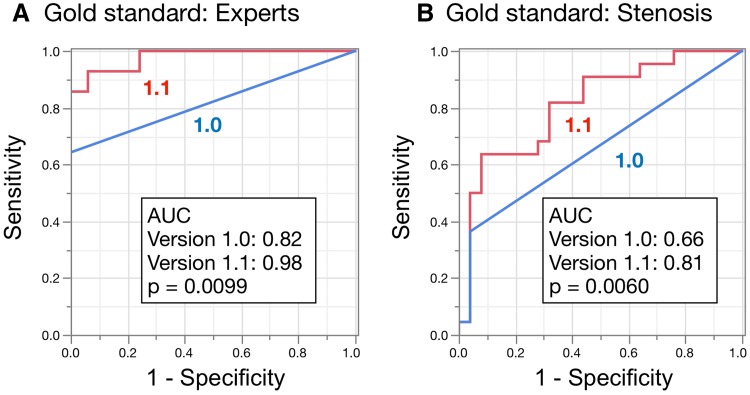
ROC curves in subset of patients with either no stenosis or single-vessel disease. Patients with revascularization and/or old myocardial infarction were excluded, and remaining 47 patients were analyzed. Gold standards were expert interpretation (**a**) and coronary stenosis (**b**). The statistical measures of sensitivity/specificity/accuracy were 64/100/89% and 93/94/94% for versions 1.0 and 1.1, respectively, with the gold standard of experts (**a**) and 37/96/68% and 64/92/79%, respectively, with that of coronary stenosis (**b**)

In addition to expert interpretation, diagnostic accuracy was assessed using coronary stenosis as another gold standard. Patients with revascularization and those with OMI were excluded from this analysis, and patients with either single-vessel disease (coronary stenosis ≥ 50%, *n* = 22) or no stenosis (*n* = 25) were included. The AUC for versions 1.0 and 1.1 were, respectively, 0.82 and 0.98 (*p* = 0.0099) when expert interpretation was the gold standard (Fig. 5a), and 0.66 and 0.81, respectively (*p* = 0.0060), when coronary stenosis was the gold standard. These findings indicate that the diagnostic accuracy of version 1.1 had improved. The statistical measures of sensitivity/specificity were 93/94% and 64/92% using the expert interpretation and coronary stenosis as the gold standard, respectively.

## Discussion

The present study showed that the diagnostic accuracy of version 1.1 was better than that of version 1.0 when assessed using the same validation database. The diagnostic accuracy was obviously improved in patients without a history of myocardial infarction or coronary revascularization (that is, without modification by therapeutic intervention).

### Computer-aided diagnosis

Visual assessment of myocardial perfusion SPECT for defects and reversibility is the initial step towards an appropriate diagnosis. Computer-assisted quantitation and evaluation play important roles in aiding visual assessment [11], and the most popular method of predicting prognosis has been defect scoring, such as SSS, SRS, and SDS using a 17- or 20-segment model [12, 13]. The amount of infarction and ischemia can also be determined by statistical analysis of the regional count distribution with assistance from normal databases fitted to a study population [14, 15]. In contrast, the ANN determined the probability of abnormalities in candidate regions based on a learning experience similar to that used to train humans, which might be related to integrated information about defect size, location, extent, severity, regional wall motion, sex, and other factors. Therefore, the ANN might mimic the learning processes through which trainees develop the diagnostic ability to become nuclear cardiology experts. The superior diagnostic accuracy of the ANN system over scoring methods has already been established [2, 3].

### Gold standard for training

The definition of a true diagnosis was based on the expert reading for both versions 1.0 and 1.1 in the present study. Since the target of the artificial intelligence applied in this study was to achieve diagnostic accuracy comparable to that of human experts, gold standards of coronary stenosis and fractional flow reserve were not applied. A gold standard comprising physicians’ readings had been implemented in a study using the PERFEX system [1]. Although the detection of (for example) anatomical stenosis might be another target of ANN training, stenosis and physiological ischemia might not be identical [16]. Therefore, if experts cannot identify abnormalities on MPI acquired from patients with triple-vessel disease, an ANN would also be unable to do so. However, even when expert interpretation is defined as truth, the improved diagnostic ability of version 1.1 represents progress and support for clinical applications associated with coronary artery disease. Nevertheless, the ability of version 1.1 to accurately diagnose single-vessel disease was improved when coronary stenosis was the gold standard.

### Improvement for detecting ischemia

The major improvement in version 1.1 was in its ability to detect stress-induced ischemia in patients without therapeutic modifications resulting from coronary intervention and without myocardial infarction. From our experience with applying ANN version 1.0, we found that small areas or slight degrees of ischemia were overlooked [3]. Therefore, during the development of the new version, we tried to select more candidate regions of abnormalities, and trained the ANN to identify minor degrees of abnormality. That is, the ANN learned to judge minor abnormalities as positive during the present training and development. The ANN was trained using supervised learning; the quality of the content that experts use to teach the ANN is an important part of software development using artificial intelligence.

Although we could not differentiate contribution of each feature in the neural network system, the integrated learning process was effective for improved diagnostic accuracy. Interestingly, intermediate ANN probability values were more often calculated for detecting ischemia by the version 1.1. Due to this change, sensitivity was improved for detecting ischemia while specificity was kept high (or low false-positive rate).

### Neural network for clinical practice

The practical method of applying the ANN to clinical practice should be considered. The relationship between ANN probability and defect scores is not linear [3, 4]. Summed stress, rest and difference scores all steeply increased when the ANN probability was > 0.80, which means that the ANN probability could play a unique role in the diagnosis of coronary artery disease. Clinical decisions as to whether or not infarction and ischemia actually exist on MPI are often borderline, and the truth is not always clear. Under such circumstances, expressing perfusion abnormalities as probabilities might be more practical than simply announcing, for example, that ischemia is suspected or cannot be denied. However, diagnostic relevance should be further investigated since such approaches are not common to medical diagnostics. Since estimated areas of ischemia vary widely among physicians, the presence of defects and ischemia suggested by appropriate software packages would help to reduce the inter-observer variability of clinical interpretations [17].

### Limitations

One limitation of the present study is that it included only 106 patients who had undergone coronary angiography. Considering that the diagnostic accuracy of version 1.1 has already been established based on 364 patients [4], the present study seems sufficiently valid for comparisons between the two versions. When patients with old myocardial infarction and post-revascularization conditions were included, truth could not be established. However, more precise analyses including follow-up and prognostic investigations might be feasible in future studies that include a sufficient number of patients.

## Conclusions

The ANN version 1.0 was retrained with a Japanese database to create version 1.1 and then compared with the original ANN version 1.0 using the same dataset. The diagnostic ability of version 1.1 was better, mainly when patients had induced ischemia without revascularization and no myocardial infarction. The new ANN version 1.1 could serve in clinical practice as a second opinion for diagnoses based on stress myocardial perfusion images.

## Abbreviations

ANN: Artificial neural network
AUC: Area under the curve
MIBI: Methoxyisobutylisonitrile
MPI: Myocardial perfusion imaging
OMI: Old myocardial infarction
ROC: Receiver-operating characteristics
SDS: Summed difference score
SRS: Summed rest score
SSS: Summed stress score
